# Feasibility of Systematic Respiratory-Gated Acquisition in Unselected Patients Referred for ^18^F-Fluorodeoxyglucose Positron Emission Tomography/Computed Tomography

**DOI:** 10.3389/fmed.2018.00036

**Published:** 2018-02-19

**Authors:** Philippe Robin, David Bourhis, Brieuc Bernard, Ronan Abgral, Solène Querellou, Alexandra Le Duc-Pennec, Pierre-Yves Le Roux, Pierre-Yves Salaün

**Affiliations:** ^1^Service de Médecine Nucléaire, EA 3878 (GETBO) IFR 148, Centre Hospitalier Régional et Universitaire de Brest, Université de Bretagne Occidentale, Brest, France

**Keywords:** positron emission tomography/computed tomography, fluorodeoxyglucose, respiratory gating, systematic acquisition without increasing acquisition time, feasibility, quantitative impact for lung or liver lesions

## Abstract

**Objective:**

Respiratory motion in ^18^F-fluorodeoxyglucose positron emission tomography/computed tomography (FDG PET/CT) induces blurred images, leading to errors in location and quantification for lung and abdominal lesions. Various methods have been developed to correct for these artifacts, and most of current PET/CT scanners are equipped with a respiratory gating system. However, they are not routinely performed because their use is time-consuming. The aim of this study is to assess the feasibility and quantitative impact of a systematic respiratory-gated acquisition in unselected patients referred for FDG PET/CT, without increasing acquisition time.

**Methods:**

Patients referred for a FDG PET/CT examination to the nuclear medicine department of Brest University Hospital were consecutively enrolled, during a 3-month period. Cases presenting lung or liver uptakes were analyzed. Two sets of images were reconstructed from data recorded during a unique acquisition with a continuous table speed of 1 mm/s of the used Biograph mCT Flow PET/CT scanner: standard free-breathing images, and respiratory-gated images. Lesion location and quantitative parameters were recorded and compared.

**Results:**

From October 1 2015 to December 31 2015, 847 patients were referred for FDG PET/CT, 741 underwent a respiratory-gated acquisition. Out of them, 213 (29%) had one or more lung or liver uptake but 82 (38%) had no usable respiratory-gated signal. Accordingly, 131 (62%) patients with 183 lung or liver uptakes were analyzed. Considering the 183 lesions, 140 and 43 were located in the lungs and the liver, respectively. The median (IQR) difference between respiratory-gated images and non-gated images was 18% (4−32) for SUVmax, increasing to 30% (14−57) in lower lobes for lung lesions, and −18% (−40 to −4) for MTV (*p* < 0.05). Technologists’ active personal dosimetry and mean total examinations duration were not statistically different between periods with and without respiratory gating.

**Conclusion:**

This study showed that a systematic respiratory-gated acquisition without increasing acquisition time is feasible in a daily routine and results in a significant impact on PET quantification. However, clinical impact on patient management remains to be determined.

## Introduction

^18^F-fluorodeoxyglucose positron emission tomography/computed tomography (FDG PET/CT) is a functional imaging method which has widely demonstrated its clinical value, especially in oncology (1). Quantitative indices in PET such as standardized uptake value (SUV) allow lesions characterization (2, 3), which can provide prognostic information or can be used for therapeutic evaluation (4, 5).

Positron emission tomography acquisition usually requires about 2–3 min for a single bed position, or about 15 min for a whole-body acquisition from the head to the upper limbs. During such an acquisition, patients cannot hold breathing for the entire duration of the acquisition. Images are, therefore, influenced by respiratory motion. The average duration of a respiratory cycle is about 5 s (6). Respiratory motion affects mostly thoracic and abdominal organs, leading to a substantial displacement of lesions during the respiratory cycle resulting in blurred images, errors in lesion location and inaccurate quantification of tracer uptake [SUVmax underestimation, and metabolic tumor volume (MTV) overestimation], especially for lung and for upper abdominal lesions (7–9).

Various respiratory gating methods have been developed to correct for these artifacts, and most of current PET/CT scanners are equipped with a respiratory gating system (10, 11). However, respiratory gating methods are mostly not routinely performed, mainly because their use is time-consuming. Indeed, for patients with known lung nodule or upper abdominal lesions, a specific respiratory gating procedure can be planned before acquisition, leading to a reasonable increase of total acquisition time. Nevertheless, for many patients, the presence of lung or upper abdominal lesions is unknown and is only discovered after completion of a whole-body acquisition. An additional dedicated respiratory-gated acquisition on chest or abdomen is thus necessary, leading to a disruption of the daily program. As a consequence, this additional respiratory-gated acquisition is, in fact, rarely performed in most of the PET centers.

An alternative approach would be to perform a systematic respiratory-gated acquisition in all scheduled patients, without increasing time acquisition. The respiratory gating system HD Chest^®^ (Siemens Medical Solutions, Erlangen, Germany) is an amplitude-based respiratory-gated method. This system allows for keeping 35% of the respiratory cycle when the amplitude of the lung motion is the lowest at its minimum. Therefore, the scan duration should be three times longer to maintain image quality. However, because of a usually high signal-to-noise ratio in the chest areas, it is conceivable that such gated image quality could be acceptable without increasing scan duration.

Thus, the aim of the study is to assess the feasibility and the quantitative impact of a systematic respiratory-gated acquisition in unselected patients referred for FDG PET/CT, without increasing acquisition time.

## Materials and Methods

### Study Design

Patients referred for a FDG PET/CT examination to the nuclear medicine department of Brest University Hospital were consecutively enrolled, during a 3-month period. Patients under 18 years old, brain studies (no whole-body scan) or head-and-neck studies (specifics acquisitions for radiotherapy planning already performed for these patients) were excluded. Cases presenting one or more lung or liver uptakes were selected and analyzed by a senior physician. All procedures performed in this study were in accordance with the ethical standards of the institutional research committee on human experimentation and with the Helsinki Declaration of 1975, as revised in 2008. Ethical review and approval was not required in accordance with the national and institutional requirements. All patients provided written informed consent.

### FDG PET/CT Acquisitions and Reconstructions

The PET/CT data were acquired using a Biograph mCT Flow (Siemens Medical Solutions, Erlangen, Germany) equipped with enhanced axial field of view (TrueV), which enables a single continuous motion of the patient table instead of performing the PET scan in combining different bed positions. Patients fasted for at least 6 h before PET acquisition, and the blood glucose level had to be less than 10 mmol/L before an injection of 3 MBq/kg of ^18^F-FDG. Intravenous injection was followed by a 60-min resting period in a quiet room before acquisition. Data were recorded in List-mode from the head to the upper limbs, synchronized with respiratory gating signal at 1 mm/s table speed. The respiratory signal was measured using a motion monitoring system with a pressure sensor which detect the external respiratory motion (pressure change) in real time (AZ-733 V; Anzai Medical Corporation, Tokyo, Japan). The belt was placed on the table prior to the installation of patients, and the technologists only strapped the belt around the patient’s chest. No specific instructions were given to patients.

Computed tomography was performed from mid-forehead to the upper limbs in normal shallow breathing using a low-dose setting (120 kVp, 100 mAs). Intravenous iodinated contrast medium was administered in patients without contraindication. Data obtained from the CT scan were used for attenuation correction of PET data and for fusion with attenuation-corrected PET images. CT slice thickness was set at 3 mm.

Data were reconstructed with TrueX algorithm (three dimensions ordered subsets expectation maximization iterative reconstruction with time of flight and point spread function compensation, 21 subsets, 2 iterations, and a 2-mm Gaussian post-filter) in 4 mm × 4 mm × 2 mm voxels. Two sets of images were reconstructed from data recorded during the acquisition. The first set consisted in standard free-breathing whole-body FDG PET/CT images. The second set corresponded to amplitude-based respiratory-gated images (HD Chest^®^ images) centered on chest and upper abdomen.

### FDG PET/CT Analysis

In order to analyze the impact of respiratory gating on image quality, we calculated the signal-to-noise ratio (SNR = SUVmean_Background_/Standard Deviation_Background_) and the contrast-to-noise ratio [CNR = (SUVmax_Lesion_-SUVmean_Background_)/Standard Deviation_Background_] in respiratory-gated and non-gated images.

Positron emission tomography/computed tomography scan were reviewed by an experience nuclear medicine physician. Lesions were defined as an increased non-physiological uptake relative to local background, with no predetermined cut-off and located in lungs or in liver.

Lesions were classified as follows: unique, multiple, or diffuse. A lesion with a focal uptake well differentiated from background with morphological abnormality was defined as unique. More than one focal uptake well differentiated from background with morphological abnormalities were defined as multiple, with a maximum of two lesions per organ as in PERCIST criteria (12). Lesions with unclear outlines were classified as diffuse (pleural effusion, diffuse secondary liver extension, pulmonary infection). Lung lesions were also recorded according to their location in the upper lobe, right middle lobe, or lower lobe.

For quantification, tumor delineation was performed using an adaptive threshold method (13). After a dedicated phantom calibration, the adaptative threshold was defined as follows: *a* + *b*/(S/B), with S/B corresponded to the signal to background ratio, i.e., the ratio between SUVmax in tumor and SUVmean in a background area near the tumor, *a* and *b* were determined by a calibration (*a* = 26.26 and *b* = 52.67).

The SUVmax and MTV were recorded for all lesions. Total lesion glycolysis (TLG), defined as the SUVmean × MTV, was calculated. All measurements were performed using MiM^®^ software (version 6.6.4, MIM Software Inc., Cleveland, OH, USA).

We assessed the impact of lesion size on SUVmax, MTV and TLG variations between respiratory-gated and non-gated images for measurable lesions.

### Daily Practice Impact

The impact of this study on the department organization was evaluated in terms of mean examination duration, including patient positioning, belt installation, and PET/CT acquisition per patient per day. Mean examination duration was measured as the time between the beginning of the first exam and the end of the last acquisition time in 1 day, divided by the number of examinations. The daily practice impact was evaluated in terms of mean technologists’ whole-body effective dose during three consecutive months with and without systematic respiratory gating. Active dosimetry was measured using EPD-Mk2 dosimeters (APVL ingénerie, Saint-Cyr-sur-Loire, France). Dosimetry was evaluated for all technologists registered in PET-CT zone in terms of total cumulative whole-body effective Dose Hp(0.07) and Hp(10) normalized by the number of examinations. The dosimetry evaluation was performed on the APVL platform (SyGID version 4, APVL ingénerie, Saint-Cyr-sur-Loire, France).

### Statistical Analysis

Signal-to-noise ratio and CNR were compared between respiratory-gated and non-gated images. SUVmax, SUVmean, and MTV were measured in the two sets of images and compared using a Wilcoxon signed-rank test. All results (SUVmax, SUVmean, MTV, and TLG) were expressed as mean values ± SD, range values (minimum–maximum) and median values [interquartile range (Q1−Q3)] of the percentage differences between respiratory-gated method and standard whole-body acquisition. A statistical Wilcoxon signed-rank test was performed to assess the significance of the differences. A Student’s *t*-test was performed to assess the significance of the differences between mean examination duration with and without HD Chest^®^. *P*-value smaller than 0.05 was considered as statistically significant in all tests.

## Results

### Patients

**Figure 1:**
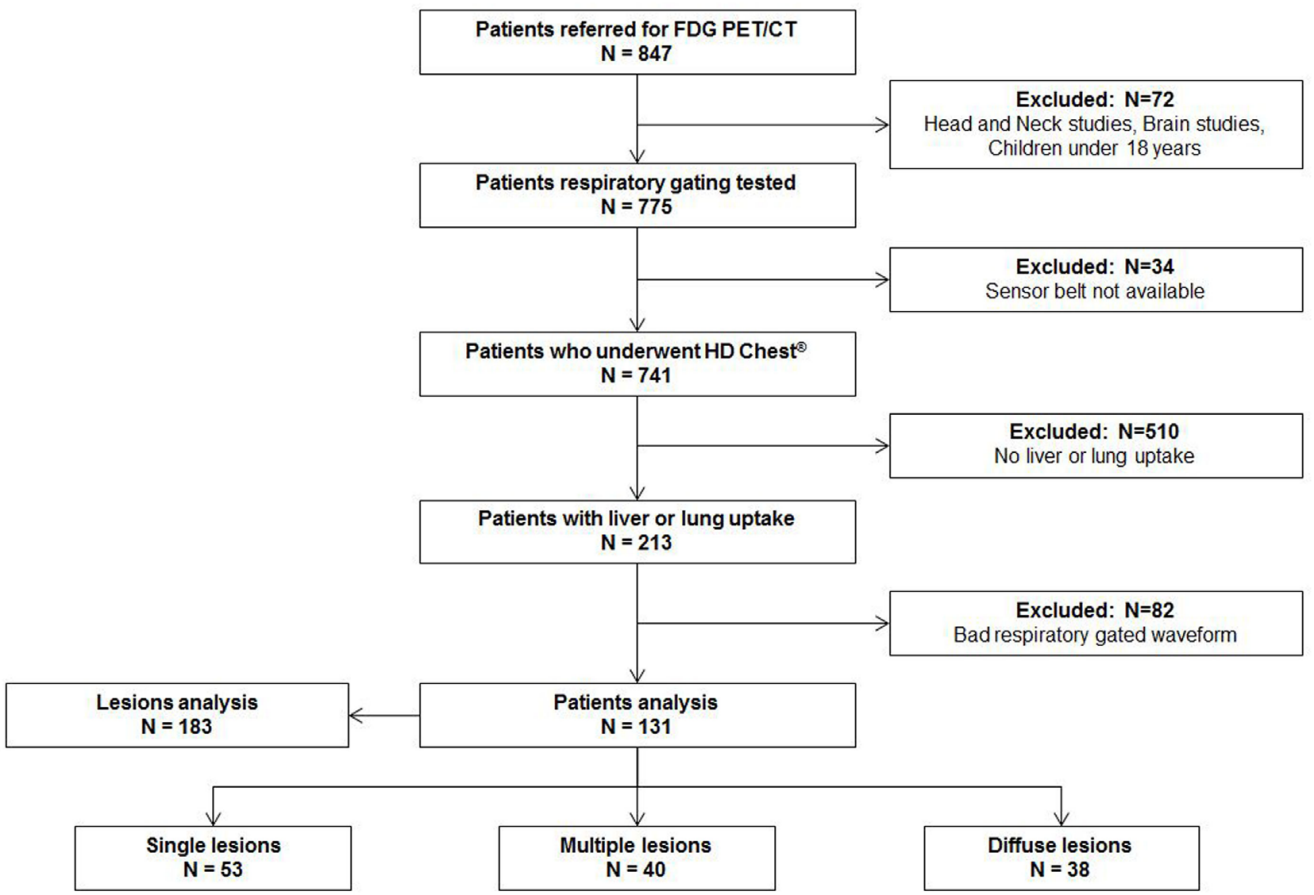
Study Flow Chart.

From October 1 2015 to December 31 2015, 847 patients were assigned for PET examinations in our nuclear medicine department. 72 (8%) patients were excluded because they had dedicated non-gated protocols (brain, head-and-neck cancer, pediatric exams). Data were not available for 34 (4%) patients because the sensor belt was not available at the beginning of the exam. Among the remaining 741 patients, 213 (29%) had one or more lung or liver uptakes. Over those 213 patients, 82 patients (38%) had a bad respiratory-gated signal (inability to breathe regularly or displacement of respiratory sensor belt during acquisition with a flat respiratory waveform). Finally, 131 (62%) patients with 183 lung or liver uptakes were analyzed. The study flow chart is presented in Figure 1. Patients characteristics, tumor characteristics, and lesions measurements (SUVmax, SUVmean, MTV) with and without respiratory gating are presented in Table 1.

**Table 1:** Patients characteristics, tumor characteristics, number of lesions per patient, and lesion location and lesions measurements [SUVmax, SUVmean, metabolic tumor volume (MTV)] with and without respiratory gating.

Age, year (mean ± SD)	65.1 ± 10.9
Male gender (%)	60
Number of patients with	
Unique lesion, *n* (%)	53 (40)
Multiple lesions, *n* (%)	40 (31)
Diffuse lesions, *n* (%)	38 (29)
Pulmonary lesions, *n* (%)	140 (77)
Lesion location	
Upper lobe	70 (50)
Middle lobe	17 (12)
Lower lobe	53 (38)
Liver lesions (%)	43 (23)
Number of patients with	
1 lesion (%)	85 (65)
2 lesions (%)	42 (32)
3 lesions (%)	3 (2)
4 lesions (%)	1 (1)
Lesions measurements [mean ± SD (min–max); median (Q1–Q3)]	
Without respiratory gating	
SUVmax	10 ± 7 [1–42]; 8 (5–14)
SUVmean	5 ± 3 [1–19]; 4 (3–6)
MTV, cm^3^	23 ± 75 [0–582]; 3 (1–9)
With respiratory gating	
SUVmax	12 ± 8 [1–42]; 11 (6–16)
SUVmean	6 ± 4 [1–20]; 5 (3–8)
MTV, cm^3^	18 ± 63 [0–583]; 2 (1–7)

### SNR and CNR in Respiratory-Gated and Non Respiratory-Gated Images

**Figure 2:**
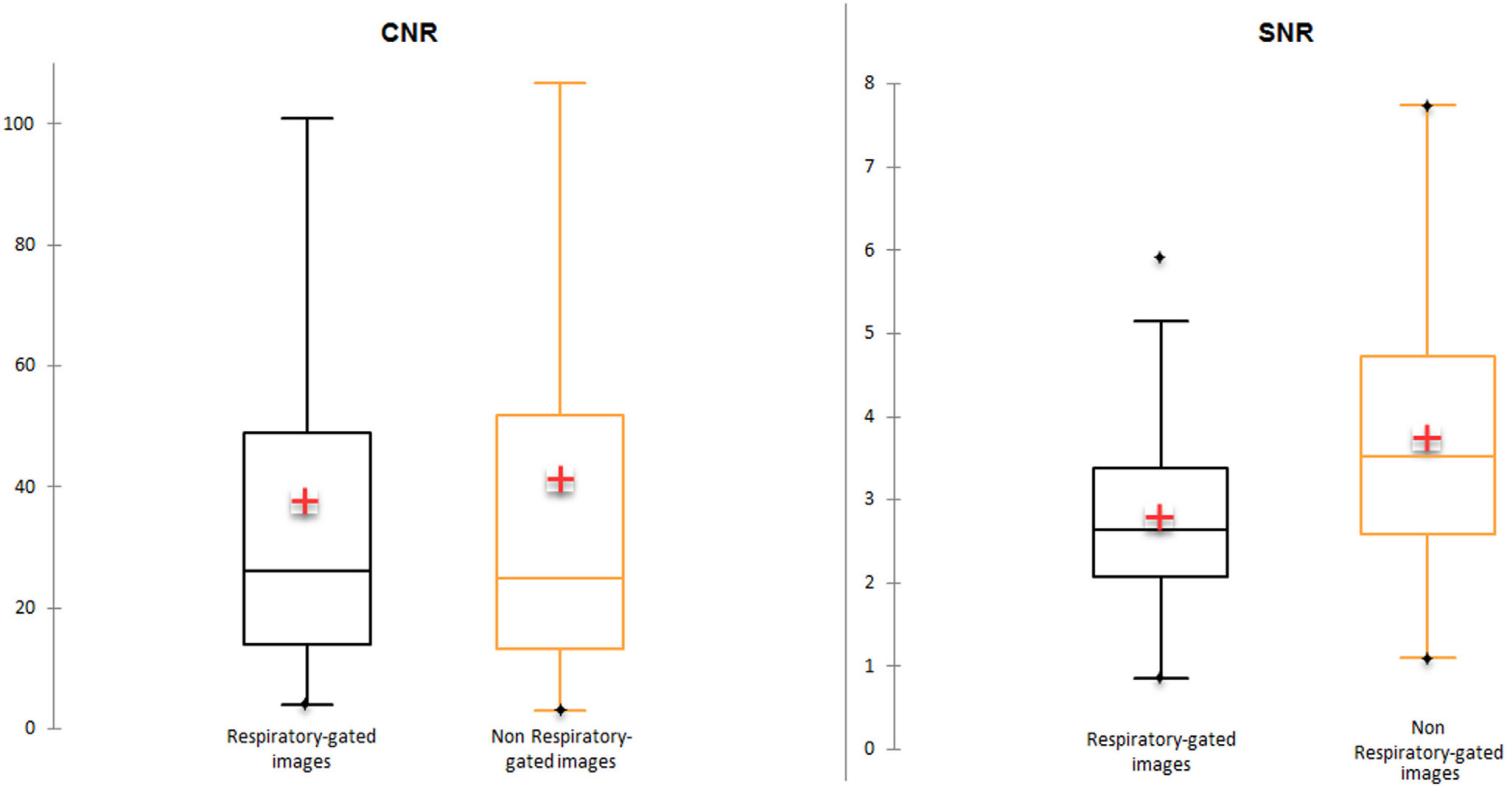
Signal-to-noise ratio (SNR) and contrast-to-noise ratio (CNR) in respiratory-gated and non-gated images.

Mean SNR and mean CNR were lower in respiratory-gated images (2.8 and 37.7 for SNR and CNR, respectively) in comparison with non-gated images (3.7 and 41.2 for SNR and CNR, respectively). Median CNR was higher in respiratory-gated images in comparison with non-gated images (26 vs. 24.9) (Figure 2).

### Lesion Analysis

**Figure 3:**
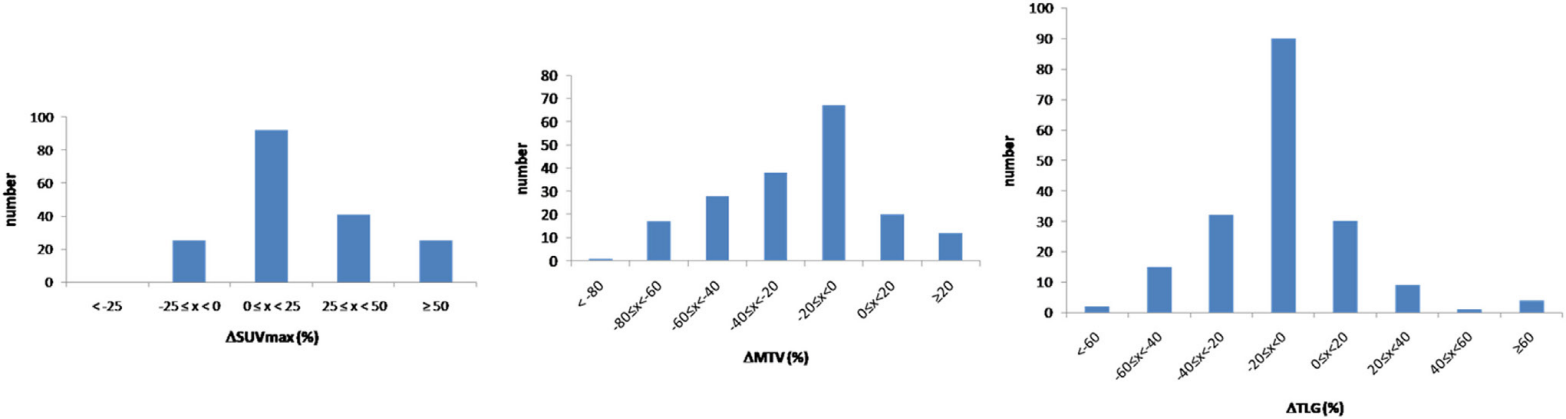
ΔSUVmax, ΔMTV, and ΔTLG ranked by increasing difference value in the population.

Considering the 183 lesions, 140 and 43 lesions were located in the lungs and in the liver, respectively. An increase of more than 25% of SUVmax between respiratory-gated and non-gated images was recorded in 66 patients (36.1%). A decrease of more than 20% of MTV between respiratory-gated and non-gated images was recorded in 84 patients (45.9%) (Figure 3).

The median difference between respiratory-gated images and non-gated images for SUVmax was 18% (4−32), for MTV was −18% (−40 to −4) and for TLG was −8% (−22 to −1) (*p* < 0.05). Considering the 140 lungs lesions, the median difference between respiratory-gated images and non-gated images for SUVmax was 15% (3−36), for MTV was −15% (−37 to −1) and for TLG was −6% (−17 to 0).

**Figure 4:**
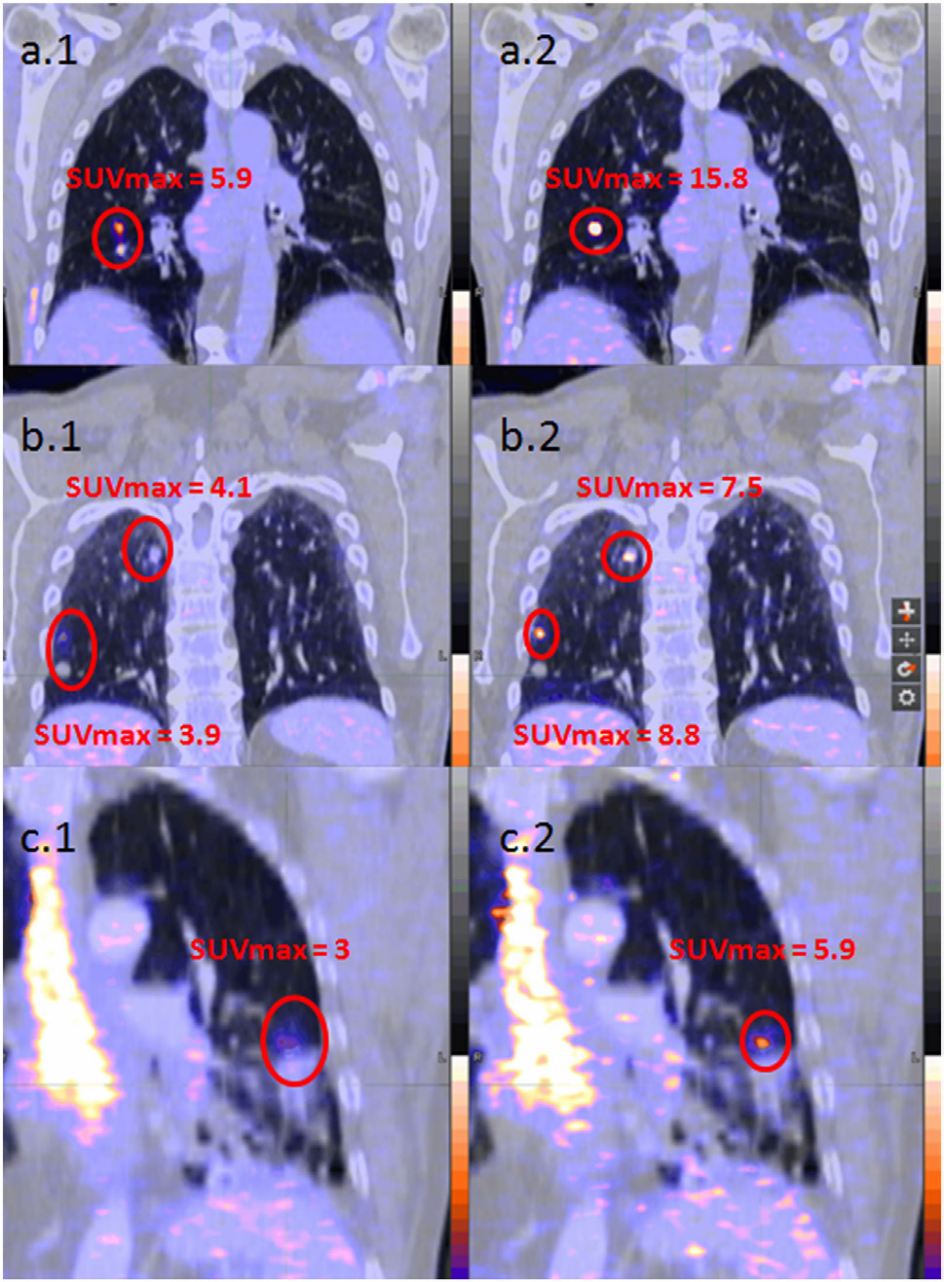
Examples of patient analysis [**(A)** unique lesion, **(B)** multiple lesions, and **(C)** diffuse lesions; 1. left images without respiratory gating; 2. right images with respiratory gating].

The median difference in lower lobes for lung lesions was 30% (14−57) for SUVmax, −28% (−52 to −14) for MTV and −11% (−25 to −2) for TLG. The median difference in upper lobes for lung lesions was 5% (0−20) for SUVmax, −7% (−22 to 2) for MTV and −4% (−13 to 0) for TLG. All results considering patients and lesion analysis are summarized in Table 2. Examples of single, multiple, and diffuse lesions are shown in Figure 4.

**Table 2:** All lesions results, lung lesions results, and patients analysis results expressed as median (Q1−Q3), and mean ± SD [min–max]; *: statistically significant difference (*p* < 0.05).

		ΔSUVmax	ΔMTV	ΔTLG
All lesions	All (*n* = 183)	18%* (4−32)	−18%* (−40 to −4)	−8%* (−22 to −1)
		23 ± 26 [−19 to 168]	20 ± 31 [−86 to 115]	−9 ± 25 [−75 to 111]
	Lungs (*n* = 140)	15%* (3 to 36)	−15%* (−37 to −1)	−6%* (−17 to 0)
		23 ± 29 [−19 to 168]	−18 ± 30 [−86 to 114]	−7 ± 23 [−71 to 97]
	Liver (*n* = 43)	20%* (13−28)	−28%* (−43 to −16)	−18%* (−29 to −4)
		21 ± 16 [−8 to 78]	−26 ± 34 [−79 to 115]	−16 ± 29 [−75 to 111]

Lung lesions	Upper Lobe (*n* = 70)	5%* (0−20)	−7%* (−22 to 2)	−4%* (−13 to 0)
		11 ± 15 [−12 to 58]	−8 ± 26 [−74 to 114]	−3 ± 21 [−58 to 97]
	Middle Lobe (*n* = 17)	17%* (2−52)	−15% (−46 to 0)	−13% (−17 to 6)
		25 ± 29 [−19 to 66]	−15 ± 39 [−65 to 67]	−1 ± 34 [−50 to 90]
	Lower lobe (*n* = 53)	30%* (14 to 57)	−28%* (−52 to −14)	−11%* (−25 to −2)
		40 ± 35 [−6 to 168]	−32 ± 27 [−86 to 36]	−15 ± 21 [−71 to 39]

Single lesions	All (*n* = 53)	20%* (4−30)	-17%* (−36 to −7)	−8%* (−22 to −2)
		25 ± 30 [−11 to 168]	−22 ± 24 [−69 to 41]	−11 ± 18 [−51 to 60]
	Lungs (*n* = 43)	16%* (4−34)	−16%* (−33 to 5)	−6%* (−16 to −2)
		24 ± 32 [−11 to 168]	−19 ± 23 [−69 to 41]	−8 ± 17 [−49 to 60]
	Liver (*n* = 10)	24%* (19−27)	−31%* (−44 to −26)	−22%* (−30 to −12)
		29 ± 19 [15−78]	−39 ± 17 [−68 to −15]	−24 ± 15 [−51 to −3]

Multiple lesions	All (*n* = 85)	16%* (3 to 34)	−19%* (−46 to 5)	−8%* (−21 to 0)
		22 ± 25 [−19 to 117]	−21 ± 32 [−79 to 115]	−9 ± 27 [−75 to 111]
	Lungs (*n* = 62)	15%* (3−38)	−15%* (−45 to −5)	−7%* (−18 to 0)
		24 ± 28 [−19 to 117]	−19 ± 29 [−74 to 67]	−7 ± 23 [−58 to 90]
	Liver (*n* = 23)	17%* (9−24)	−23%* (−45 to −16)	−15%* (−30 to −1)
		18 ± 14 [−5 to 48]	−24 ± 40 [−79 to 115]	−16 ± 36 [−75 to 111]

Diffuse lesions	All (*n* = 45)	19%* (3−31)	−15%* (−33 to 0)	−6% (−23 to 0)
		22 ± 26 [−14 to 110]	−15 ± 36 [−86 to 114]	−8 ± 28 [−71 to 97]
	Lungs (*n* = 35)	14%* (3−38)	−13%* (−27 to 0)	−3%* (−17 to 1)
		22 ± 29 [−14 to 110]	−14 ± 38 [−86 to 114]	−7 ± 30 [−71 to 97]
	Liver (*n* = 10)	28%* (20−30)	−27%* (−39 to −6)	−18%* (−26 to −3)
		22 ± 14 [−8 to 39]	−19 ± 29 [−51 to 49]	−11 ± 22 [−33 to 38]

**Figure 5:**
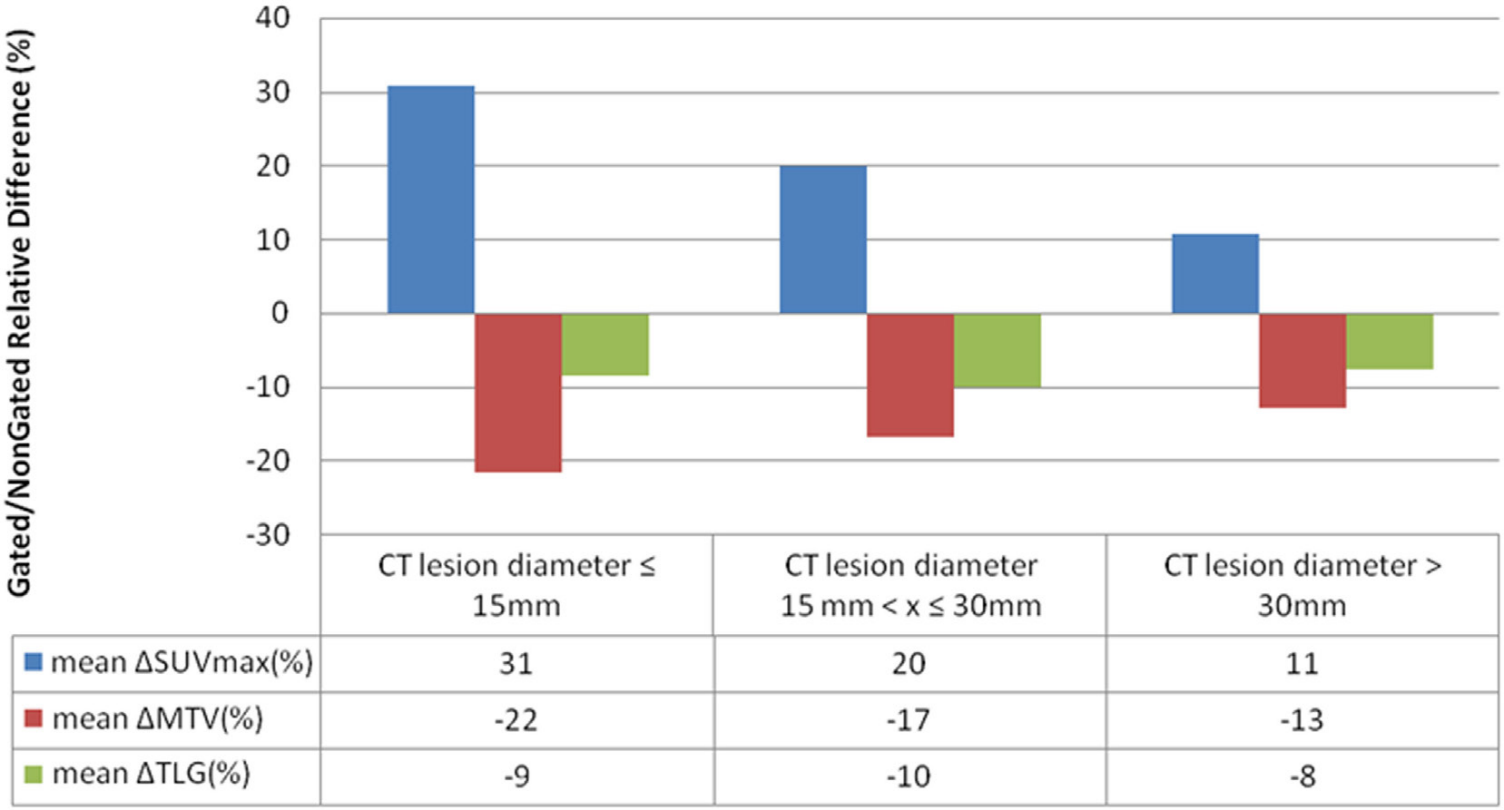
Mean SUVmax, metabolic tumor volume (MTV) and total lesion glycolysis (TLG) variations between respiratory-gated images and non-gated images according to lesions size for 119 measurables lesions.

SUVmax, MTV, and TLG variations between respiratory-gated and non-gated images were the highest for lesions smaller than 15 mm in comparison with lesions larger than 15 mm. SUVmax, MTV, and TLG variations were small for lesions bigger than 30 mm (Figure 5).

**Figure 6:**
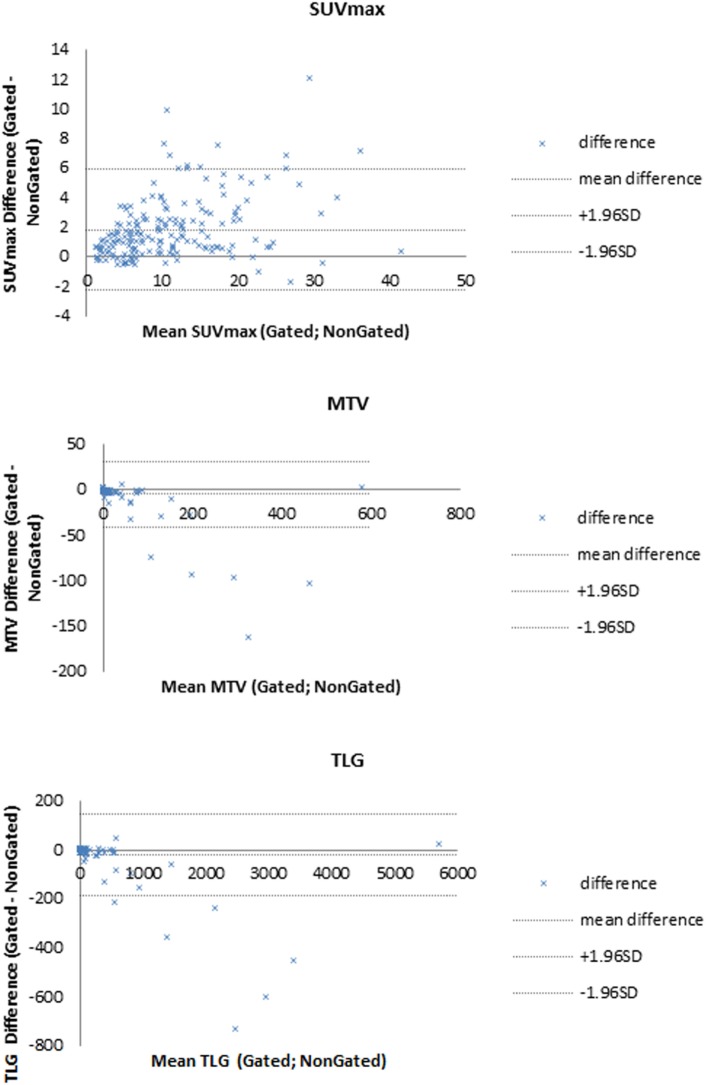
Bland–Altman plots for SUVmax, metabolic tumor volume (MTV), and total lesion glycolysis (TLG).

Bland-Altman plots for SUVmax, MTV, and TLG measurements are presented Figure 6.

### Daily Practice Impact

The technologists’ active personal dosimetry in PET–CT zone, measured in three consecutive months (58 days) with respiratory-gated acquisitions (2015-10-01 to 2015-12-31) vs. without respiratory-gated acquisitions (2015-01-01 to 2015-03-31) was 5,968 vs. 6,132 µSv for Hp(0.07), and 5,629 vs. 5,748 µSv for Hp(10). Normalized by the number of scans during the same period, doses were 3.4 vs. 3.7 μSv/scan for Hp(0.07), 3.2 vs. 3.4 μSv/scan for Hp(10). Considering the mean examination duration, mean respiratory-gated examinations duration was 26.9 min/examination and mean non-gated examinations duration was 27 min/examination. No significant difference was found (*p* = 0.8).

## Discussion

This study showed that a systematic respiratory-gated acquisition is feasible in a clinical context in consecutive patients referred for a whole-body FDG PET/CT, without increasing acquisition time and examination duration, and with a significant impact on quantification. Indeed in our study, over the 741 patients who underwent a systematic respiratory-gated acquisition, 18% of them (131/741) presented lung or liver lesions with a statistically significant increase in SUVmax [median increase of 18% (4–32)] measured from the respiratory-gated images in comparison with non-gated images.

These results are consistent with previous studies assessing respiratory gating. Indeed, authors reported an increase of the value of SUVmax ranging from 7 to 159% for lung lesions, especially for lung cancer (14–18). In our study, increase value of SUVmax ranged from −19 to 168%. Moreover, Van Der Gucht et al. showed that amplitude-based respiratory gating method is feasible to increase detectability and quantification of upper abdominal lesions in comparison with the conventional standard whole-body acquisition with a mean increase of 24% ± 46 in SUVmax in 31 patients enrolled with liver lesions (19). In comparison, our results showed a mean increase of 23% ± 26 in 131 patients for all lesions, and mean increase of 21% ± 16 in 43 liver lesions.

Anatomical location (upper or lower lobes) has an impact on lesion quantification. Quantitative parameters are especially modified when lesions are located in the lower lobes, in which respiratory movement is the most important. In our study, changes in SUVmax and MTV were largest for lesions in lower lobes (median increase of 30% for SUVmax, and median decrease of 28% for MTV). Indeed, it has been well established that the magnitude of structure motion is dependent on anatomical location within the lungs. Lung tumors have been reported to exhibit motion up to >3 cm in the cranio-caudal direction during normal respiration, while others move only a few millimeters or not at all (20). Similarly, Grootjans et al. found large changes in SUVmean and in lesion volume using an amplitude-based respiratory gating system in patients with primary lung cancer for lesions located in the middle and lower lobes (21).

Respiratory-gated could have a potential clinical impact on patient management especially in therapeutic evaluation. A threshold of 25% for the variation of the SUVmax is usually used to differentiate progression disease, stable disease or partial response in EORTC criteria: an increase of SUVmax greater than 25% is considered as a metabolic progression; an increase in the SUVmax of less than 25% or a decrease of less than 25% is considered as stable disease; partial metabolic response is classified as a decrease of max SUV greater than 25% (22). Our results showed that respiratory-gated images provided a median increase of 30% (14−57) for SUVmax in lower lung lobes. Indeed, a lung lesion could have been considered as a disease progression by increase of more than 25% of the SUVmax solely because of the use of respiratory-gated images.

In addition, previous studies have demonstrated that SUVmax could be a prognostic factor in cancer patients. Sasaki et al. found that SUVmax was an independent prognostic factor of overall survival and progression-free survival, with a threshold of 5 in patients in non-small cell lung cancer (23). In our study, 10 patients would have been considered with a good prognosis (SUVmax <5) in non-gated images, and with a poor prognosis (SUVmax >5) in gated images.

Other studies have demonstrated that the MTV was a more useful tool for the characterization of lesions. MTV is not only based on a single pixel as the SUVmax but over a larger region of interest within the tumor. Indeed several studies have shown that the MTV was an independent prognostic factor of overall survival especially in locally advanced cancers of the upper aerodigestive tract (24, 25). Our results showed that respiratory-gated images provided a median decrease of 18% (−40 to −4) for MTV.

The major limitation of the adaptive threshold methods is the dependence on optimization using phantom acquisitions for calibration. However, to date, there are no clear guidelines regarding the automatic delineation of PET functional volume.

The technologists’ active personal dosimetry in PET–CT zone during this 3-months study was not higher compared to personal dosimetry recorded in three consecutive months during another period without any respiratory-gated acquisitions (5,968 vs. 6,132 µSv for Hp0.07, 5,629 vs. 5,748 µSv for Hp10). Considering the mean examination duration, no significant difference was found between those two periods. Indeed the use of systematic respiratory-gated method leads neither to an increase of radiation exposure for nuclear medicine technologist nor to a raise of examination duration. The technologist’s workflow was only modified by positioning the belt on the table and then around the patient’s chest.

One limitation about this kind of respiratory gating is that it only concerns PET data. CT scans are performed without respiratory gating with free breathing, a spatial mismatch between the PET and CT images could exist, especially for lesions in lower lobes which exhibit a large displacement during the respiratory cycle. Spatial mismatch between PET and CT images results in inappropriate attenuation correction and inaccuracies in quantification. Respiratory-gated CT could be proposed but increasing radiation exposure (26). Another limitation is that 82 patients (38%) had a bad respiratory-gated waveform due to either inability to breathe regularly, or displacement of the pressure sensor belt during acquisition and, thus, were excluded from analysis. The high proportion of bad respiratory-gated signal can be improved by technologist’s training in positioning sensor belt around the chest (i.e., lower ribs), as well as a more frequently sensor calibration of the system. On the other hand, lesion characterization or therapeutic evaluation could have modified in the remaining 131 patients (62%) who underwent respiratory gating, without increasing technologist radiation dose or impacting daily organization. This limitation might probably be reduced by technologist training. Moreover, a dedicated respiratory-gated acquisition could then be performed after the examination in case of failure.

The Biograph mCT Flow (Siemens Healthcare, Erlangen, Germany) is the first PET/CT system that moves the patient through the gantry while continuously acquiring PET data, instead of using sequential static acquisitions (i.e., stop-and-go acquisition mode). On the one hand, the FlowMotion technology allows physicians to adjust respiratory motion management to the dimensions of organs instead of two or three bed positions with standard acquisition. On the other hand, the FlowMotion technology eliminates the need to overlap scan positions and enables noise uniformity to the edge of the scan range as the patient is continuously moved through the system.

As a conclusion, this study showed that a systematic respiratory-gated acquisition without increasing acquisition time is feasible in daily routine in consecutive unselected patients referred for a whole-body FDG PET/CT and involved a significant impact on PET quantification (SUVmax and MTV). Moreover, technologists’ active personal dosimetry and total examination durations were not higher with respiratory gating than without respiratory gating. However, clinical impact on patient management remains to be determined.

## Ethics Statement

All procedures performed in this study were in accordance with the ethical standards of the institutional research committee on human experimentation and with the Helsinki Declaration of 1975, as revised in 2008. Ethical review and approval was not required in accordance with the national and institutional requirements. All patients provided written informed consent.

## Author Contributions

DB, PR, and P-YS had full access to all of the data in the study and take responsibility for the integrity of the data and the accuracy of the data analysis. Study concept and design: DB, P-YR, PR, and P-YS. Acquisition, analysis, or interpretation of data: all the authors. Drafting of the manuscript: DB, P-YR, PR, and P-YS. Critical revision of the manuscript: all the authors. Statistical analysis: DB. Study supervision: P-YR, P-YS.

## Conflict of Interest Statement

P-YS received a research grant from Siemens Molecular Imaging. The other authors declare that they have no conflict of interest. The reviewer AS and the handling editor declared their shared affiliation.

## Abbreviations

FDG PET/CT, ^18^F-fluorodeoxyglucose positron emission/computed tomography; MTV, metabolic tumor volume; SUV, standardized uptake value; TLG, total lesion glycolysis.
